# Visualization of VirE2 protein translocation by the *Agrobacterium* type IV secretion system into host cells

**DOI:** 10.1002/mbo3.152

**Published:** 2013-12-27

**Authors:** Philippe A Sakalis, G Paul H van Heusden, Paul J J Hooykaas

**Affiliations:** Institute of Biology, Leiden UniversitySylviusweg 72, Leiden, 2333 BE, The Netherlands

**Keywords:** *Agrobacterium tumefaciens*, BiFC, protein translocation, *Saccharomyces cerevisiae*, split GFP, type 4 secretion system, VirE2.

## Abstract

Type IV secretion systems (T4SS) can mediate the translocation of bacterial virulence proteins into host cells. The plant pathogen *Agrobacterium tumefaciens* uses a T4SS to deliver a VirD2-single stranded DNA complex as well as the virulence proteins VirD5, VirE2, VirE3, and VirF into host cells so that these become genetically transformed. Besides plant cells, yeast and fungi can efficiently be transformed by *Agrobacterium*. Translocation of virulence proteins by the T4SS has so far only been shown indirectly by genetic approaches. Here we report the direct visualization of VirE2 protein translocation by using bimolecular fluorescence complementation (BiFC) and Split GFP visualization strategies. To this end, we cocultivated *Agrobacterium* strains expressing VirE2 tagged with one part of a fluorescent protein with host cells expressing the complementary part, either fused to VirE2 (for BiFC) or not (Split GFP). Fluorescent filaments became visible in recipient cells 20–25 h after the start of the cocultivation indicative of VirE2 protein translocation. Evidence was obtained that filament formation was due to the association of VirE2 with the microtubuli.

## Introduction

The gram-negative plant pathogen *Agrobacterium tumefaciens* provokes crown gall tumor formation in dicotyledonous plant species by their genetic transformation with a set of oncogenic genes. The genes that are transferred originate from the Transfer region (T-region) of the tumor-inducing plasmid (Ti plasmid), which is present in virulent strains of this bacterium.

The DNA is translocated in a single-stranded form (T-strand) from the pathogen into the host cells. After entry into the nucleus, the T-strand is converted in a double-stranded form (T-DNA) and integrated into the host chromosomal DNA. Expression of the T-DNA genes in plant cells leads to the synthesis of plant hormones causing uncontrolled cell proliferation and results in crown gall tumor formation (Gelvin 2003).

Under conditions that induce the virulence system *A. tumefaciens* is also able to transform non-plant organisms such as yeast and fungi (Bundock et al. 1995; Bundock and Hooykaas 1996; Piers et al. 1996; De Groot et al. 1998). The ability of *A. tumefaciens* to genetically modify plants and fungi is now widely used in research and in biotechnology. For many plant and fungal species *Agrobacterium*-mediated transformation (AMT) has become the preferred method of transformation.

The bacterium uses a type IV secretion system (T4SS), encoded by *virB* genes on the Ti plasmid, for the translocation of the T-strand into host cells (Christie et al. 2005; Alvarez-Martinez and Christie 2009; Wallden et al. 2010). The coupling protein VirD4 plays an important role in substrate recognition (Cabezón et al. 1997). A number of other virulence (Vir) proteins including the relaxase VirD2 play a role inside the bacterium in the formation of the T-strand (Ward and Barnes 1988), whereas other virulence proteins are translocated into the host cells to assist in the transformation process and are nowadays often referred to as effector proteins. These latter proteins, which include VirF can be expressed in plant cells to complement mutant bacteria (Regensburg-Tuïnk and Hooykaas 1993). More evidence for virulence protein translocation by the *Agrobacterium* T4SS was obtained by the development and use of the CRAfT assay (Vergunst et al. 2005). In this assay, virulence proteins are fused at their N-termini to the Cre recombinase. Virulence protein translocation can subsequently be detected by monitoring for a Cre-mediated recombination event in the plant or yeast host cells (Vergunst et al. 2000; Schrammeijer et al. 2003). By such experiments it was shown that the translocation signal of the translocated Vir proteins is an arginine-rich peptide, located at the C-termini of the virulence or effector proteins (Vergunst et al. 2005). Five virulence proteins, VirD2, VirD5, VirE2, VirE3, and VirF are translocated to plant and yeast cells independently of the T-strand. The relaxase VirD2 makes nicks at the ends (border repeats) of the T-region and becomes covalently linked to the T-strand that is subsequently formed (Ward and Barnes 1988). This VirD2-T-strand complex is translocated into host cells, but in the absence of a T-region VirD2 can be translocated to host cells also independently. This has led to the idea that DNA translocation starts with the recognition and delivery of the relaxase, which thus acts as a pilot protein. Evidence for this was obtained by deleting all but the relaxase domain of VirD2, which renders the bacterium avirulent. However, addition of the C-terminal translocation peptide of one of the other translocated virulence proteins restored virulence (van Kregten et al. 2009). It has been shown that VirE2 is a single-stranded DNA-binding protein (Gietl et al. 1987), which binds cooperatively irrespective of the sequence (Citovsky et al. 1989; Sen et al. 1989; Grange et al. 2008). It is thought that in the plant host cells the translocated T-strand with VirD2 attached interacts with translocated VirE2 virulence proteins to form a T-complex. When unbound to the T-strand, VirE2 preferentially binds to other VirE2 proteins forming an aggregate. Inside *Agrobacterium* VirE1, which is not translocated to the host cells, prevents this aggregation and also inhibits the premature binding of VirE2 to the T-strand (Sundberg and Ream 1999; Dym et al. 2008). Binding of VirE2 to the T-strand prevents degradation by nucleases in the host cell (Rossi et al. 1996). The VirE2 protein together with VirD2 mediates the nuclear uptake of the T-complex (Zupan et al. 1996; Ziemienowicz et al. 2001).

In plants, VIP1 (VirE2 interacting protein 1) contributes to VirE2 nuclear translocation and tumorigenicity (Tzfira et al. 2001). This host protein is a transcription factor which is phosphorylated during infection and then directed toward the nucleus to activate the expression of pathogenesis-related genes (Djamei et al. 2007). In the nucleus, VIP1 binds to promoters of genes with a VIP1-responsive element (VRE), which leads to their transcription (Lacroix and Citovsky 2013).

VirE3 is a nuclear protein that associates with the general transcription factor pBrp in plant cells and can act as a transcriptional activator (Garcia-Rodriguez et al. 2006). It has been suggested that VirE3, similar to VIP1, may facilitate nuclear import of VirE2 (Lacroix et al. 2005).

Besides *Agrobacterium* a large number of other pathogens including the human pathogens *Helicobacter pylori*, *Bartonella henselae*, and *Legionella pneumophila* employ a T4SS to translocate virulence proteins into host cells (Nagai and Roy 2003; Segal et al. 2005; Backert and Meyer 2006; Dehio 2008). The *Agrobacterium* CRAfT system has been used successfully to identify effector proteins from other species indicating that heterologous translocation signals can be recognized in some cases by the *Agrobacterium* T4SS (Hubber et al. 2004; Lin et al. 2007), but in other cases the CRAfT assay was adapted for the specific pathogen (Luo and Isberg 2004). Long lists of candidate effector proteins have also been assembled by bioinformatics approaches using the common properties of the known effector proteins or the encoding genes (Burstein et al. 2009; Chen et al. 2010; Huang et al. 2011). Translocation of candidate effector proteins can now be tested by CRAfT or by enzymatic assays that were developed to this end. Fusion to adenylate cyclase or preferably to TEM1 *ß*-lactamase allows following translocation by the detection of the respective enzymatic activity in host cells (de Felipe et al. 2008).

Development of visualization techniques applying autofluorescent proteins have resulted in many new insights in biological processes (Shaner et al. 2005). Fluorescently labeled virulence proteins have been (transiently) expressed in host cells to study their localization and function. However, the localization may differ when the effector proteins are translocated through the T4SS as the amounts may differ and by presence of other effector proteins that are translocated during infection and that may influence each other's localization. Direct visualization of effector protein translocation is hampered by the observation that effector proteins fused to green fluorescent protein (GFP) cannot be translocated through the T4SS due to the bulky nature of the fluorescent protein. In this study, we developed novel strategies based on bimolecular fluorescence complementation (BiFC) and split GFP for the direct visualization of protein translocation by the T4SS (Ciruela 2008). The split GFP approach was developed by Van Engelenburg and Palmer (2010) for the imaging of effector protein secretion by the type III Secretion System (T3SS) of *Salmonella enterica* into human cells and adapted by us for use with the T4SS. When we cocultivated *Agrobacterium* strains expressing VirE2 tagged with one part of a fluorescent protein with host cells expressing the complementary part, either fused to VirE2 (for BiFC) or not (Split GFP) fluorescent filaments became visible in recipient cells 20–25 h after the start of the cocultivation indicative of VirE2 protein translocation by the T4SS. By confocal microscopy protein translocation could be followed in real time.

**Figure 1:**
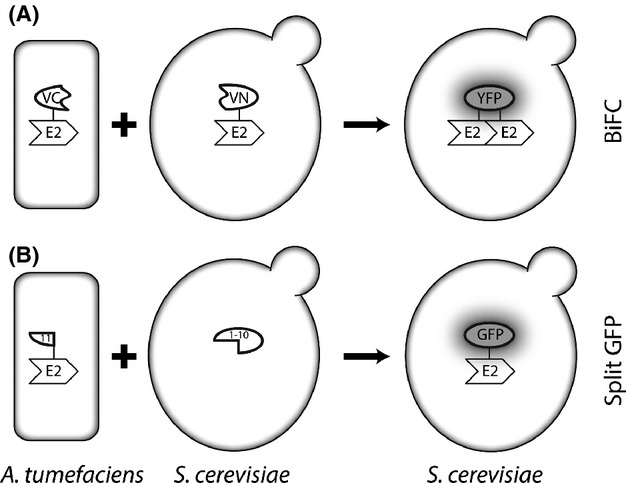
Schematic representation of the strategy to visualize translocation of VirE2 from *Agrobacterium* to yeast using BiFC and split GFP. *Agrobacterium tumefaciens* expressing VirE2 N-terminally fused to a fragment of the YFP analog Venus is cocultivated with *Saccharomyces cerevisiae* expressing VirE2 fused to the com-plementary fragment of Venus. Upon translocation of the tagged VirE2 from *A. tumefaciens* to *S. cerevisiae* it binds to the tagged VirE2 expressed in yeast resulting in reconstitution of Venus and yellow fluorescence.

To visualize translocated VirE2 proteins we made use of their self-associating properties in the absence of its chaperone VirE1 (Frenkiel-Krispin et al. 2007). Therefore, *Agrobacterium* expressing VirE2 tagged with a fragment of the yellow fluorescent protein (YFP) analog Venus was cocultivated with yeast ectopically expressing VirE2 tagged with the complementary fragment of Venus (Fig. 1). As VirE2 self-associates, fluorescent Venus is restored upon translocation of VirE2 to the yeast cell. In this way fluorescence is not seen in donor or recipient, but only after VirE2 translocation from donor into the recipient. Thus, we were able to visualize VirE2 translocation within a time frame of about 24 h after the initiation of the cocultivation. Translocated VirE2 as well as ectopically expressed tagged VirE2 were present in filamentous structures coinciding with the microtubules.

## Materials and Methods

### Yeast strains and media

Yeast strains used in this study are listed in Table S1. All yeast strains were grown in yeast extract peptone dextrose (YPD) medium or selective minimal yeast (MY) medium supplemented, if required, with histidine, leucine, tryptophan, and/or uracil to a final concentration of 20 mg/L (Zonneveld 1986). Yeast transformations were performed using the LiAc method (Gietz et al. 1995). Yeast strains carrying plasmids were obtained by transforming parental strains with the appropriate plasmids followed by selection for histidine, leucine, and/or uracil prototrophy. The integrative plasmid pRS305-GFP_1-10_ was used to transform strain CEN.PK2-1C to generate strain 426::GFP_1-10_ after selection for leucine prototrophy. Polymerase chain reaction (PCR) analysis on genomic DNA from strain 426::GFP_1-10_ with primer pair Leu2 1A and Leu2 1S and with primer pair Leu2 2A and Leu2 2S generated DNA fragments of 2399 bp and 2724 bp, respectively, indicating correct integration of pRS305-GFP_1-10_. An additional PCR to detect the presence of the sequence coding for GFP 1-10 in the yeast strain was performed with *Xba*I-GFP_1-10_-Fw and *Xho*I-GFP_1-10_-Rev.

### *Agrobacterium* strains and media

*Agrobacterium tumefaciens* strains used in this study are listed in Table S2. All *A. tumefaciens* strains were grown in LC medium (10 g/L tryptone, 5 g/L yeast extract and 8 g/L NaCl) containing (if required) the appropriate antibiotics at the following concentrations: rifampicin, 20 *μ*g/mL; gentamicin, 40 *μ*g/mL; kanamycin, 100 *μ*g/mL. *Agrobacterium tumefaciens* strains carrying plasmids were obtained by electroporation as described by den Dulk-Ras and Hooykaas (1995).

### Plasmid constructions

Plasmid constructions are described in the supporting information (Data S1). All plasmids used and constructed in this study are listed in Table S3. Cloning steps were performed in *E. coli* strain DH5*α*. PCR amplifications were done with Phusion™ High*-*Fidelity DNA Polymerase (Thermo Scientific, Landsmeer, the Netherlands) and Table S4 lists all primers used for PCR amplifications.

### Tobacco plant lines and media

*Nicotiana tabacum* SR1 plants were genetically transformed by means of leaf disk transformation according to the protocol of Sparkes et al. (2006). To obtain SR1 lines expressing GFP 1-10 we performed the leaf disk transformation with *Agrobacterium* strain AGL1(pCambia1302-GFP_1-10_) and selected for Hygromycin (50 *μ*g/mL) resistance. Transformation rendered 39 calli growing from the leaf disks. After transfer of the calli to shoot induction medium (IM), four shoots were selected and grown to full plants. Genomic DNA was isolated from the plants and integration of the T-DNA with the coding DNA sequence of GFP 1-10 was checked by PCR using primers *Xba*I-GFP_1-10_-Fw and *Xba*I-GFP_1-10_-Rev. Expression of GFP 1-10 was confirmed by Western blotting. Homozygous plants were selected for Agroinfiltration experiments.

### *Agrobacterium* yeast cocultivations

Cocultivations of *A. tumefaciens* strains and yeast recipient strains were performed as previously described (Bundock et al. 1995) with some minor modifications. After overnight growth at 29°C in LC medium supplemented with the appropriate antibiotics (Table S2), *Agrobacterium* cells were diluted to an OD_600_ ˜0.25 in IM and grown at 28°C for 6 h. In experiments using non-integrative vector-containing yeast strains, yeast was grown overnight in MY medium supplemented with the appropriate nutrients and cells were diluted fivefold in fresh MY medium supplemented with the appropriate nutrients. After growing for 6 h, yeast cells were washed with 1/10 volume of IM (without glucose) and diluted to an OD_600_ ˜0.50 in IM (without glucose) supplemented with the appropriate nutrients. Fifty microliter aliquots of the *Agrobacterium* yeast mixture were spotted on cellulose nitrate filters (Sartorius) on IM plates and these plates were left standing for 30 min before incubation at 21°C.

### Agroinfiltration

*Agrobacterium* strains were grown overnight at 29°C. After dilution to an OD_600_ ˜0.8 in IM + 200 *μ*mol/L acetosyringone [AS]), 10 mL cultures were grown for 3 h at 28°C. Subsequently the cultures were transferred into a blunt-tipped plastic 10 mL syringe (Nissho NIPRO Europe N.V., Zaventem, Belgium)) and injected into the leaves of the transgenic *N. tabacum* SR1 line expressing GFP 1-10. This was done by applying the tip of the syringe to the lower surface of the leaves and injecting with gentle pressure. Young leaves of three to 4-week old plants were used for Agroinfiltrations. After 20–24 h the lower side of the injected leaf was imaged by confocal microscopy.

### Protoplast transformation

*Arabidopsis thaliana* Columbia protoplasts were obtained from cell suspension cultures that were propagated as described by Schirawski et al. (2000). Polyethyleneglycol (PEG)-mediated transformations of protoplasts with 10 *μ*g of plasmid DNA were performed as reported by Schirawski et al. (2000). Protoplasts were imaged by confocal microscopy 24 h after transfection.

### Confocal microscopy

Yeast cells were grown in MY medium supplemented with the appropriate nutrients. All microscopic analyses were done with confocal laser scanning microscopy (CLSM) with a Zeiss Imager and Zeiss observer (Zeiss, Oberkochen, Germany), both equipped with an LSM 5 Exciter, using a 63× magnifying objective (numerical aperture 1.4). Cyano fluorescent protein (CFP) signal was detected using an argon 458 nm laser and a 475–515 nm band pass filter. GFP signal was detected using an argon 488 nm laser and a 505–530 nm band pass filter. To detect YFP signal and reconstituted BiFC signal an argon 514 nm laser and a 530–600 nm band pass filter were used. Chlorophyll fluorescence was captured using a longpass 650 nm emission filter after excitation at 488 nm. Samples for microscopic analyses of cocultivations were prepared by taking aliquots of *Agrobacterium* yeast mixtures from cellulose nitrate filters and transferring them to a cover slide in 5 *μ*L fresh IM supplemented with the appropriate nutrients. A coverslip was placed on top and the sample was sealed with transparent nail polish to prevent drying. Time-lapse experiments were performed with sealed samples. Microscopic images were analyzed using ImageJ software (Abramoff et al. 2004) and assembled using Adobe Photoshop CS4 and Adobe Illustrator CS4.

### Förster resonance energy transfer

All yeast strains were grown overnight in MY medium supplemented with appropriate nutrients. Microscopy was performed as described above. Förster resonance energy transfer (FRET) studies (Forster 1948) on the interaction between VirE2 and Tub1p were done with a sensitized emission FRET approach. For this goal, we used yeast strains 426-34Turquoise/36YFP (negative control) and 426::Turquoise-TUB1/34YFP-VirE2. Microscopic images were processed with ImageJ software, using the FRET and colocalization analyzer plugin (Hachet-Haas et al. 2006) to measure sensitized emission FRET and to calculate the FRET index. Bleed through (BT) values were calculated with the plugin using images of yeast 426-34Turquoise-VirE2 (donor BT control) and 426-36YFP-VirE2 (acceptor BT control). Using these yeast strains, we obtained a mean donor BT value of 0.164 and a mean acceptor BT value of 0.375. The relative colocalized FRET index was calculated by dividing the colocalized FRET index by 20% of the pixel values from corresponding donor fluorescence images.

### Flow cytometry

All yeast strains were grown in MY medium supplemented with the appropriate nutrients and diluted 10-fold before flow cytometry. The Guave Easycyte™ system from Merck MILLIPORE (Billerica, MA) was used and data were analyzed with CytoSoft™ software (Merck Millipore, Billerica, MA). A 488 nm laser and a 510–540 nm band pass filter were used to detect fluorescence.

## Results

### VirE2 ectopically expressed in yeast colocalizes and physically interacts with microtubules

We have shown before that the T4SS of *Agrobacterium* can translocate the effector proteins not only to plant cells but also to yeast and fungi (Schrammeijer et al. 2003). Because of its ease of handling, genetic versatility and lack of autofluorescence as compared with plant cells we initially focused on yeast as a recipient in the development of methodology for direct visualization of T4SS mediated effector translocation. We used the virulence protein VirE2 in our studies because of its abundance and because it has been shown that it self-associates in the absence of its chaperone VirE1, which is present in *Agrobacterium*, but which is not translocated into host cells.

**Figure 2:**
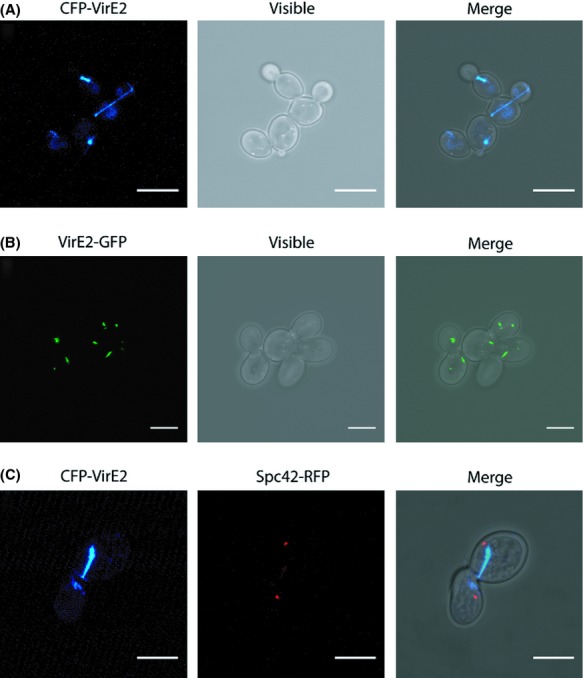
Confocal laser scanning microscopy of yeast strains 428-34CFP[VirE2] expressing CFP-VirE2 (A), of yeast strain 428-35[VirE2] expressing VirE2-GFP (B) and of 284-34CFP-VirE2 expressing CFP-VirE2 and Spc42-RFP (C). Scale bars: 7 *μ*m.

**Figure 3:**
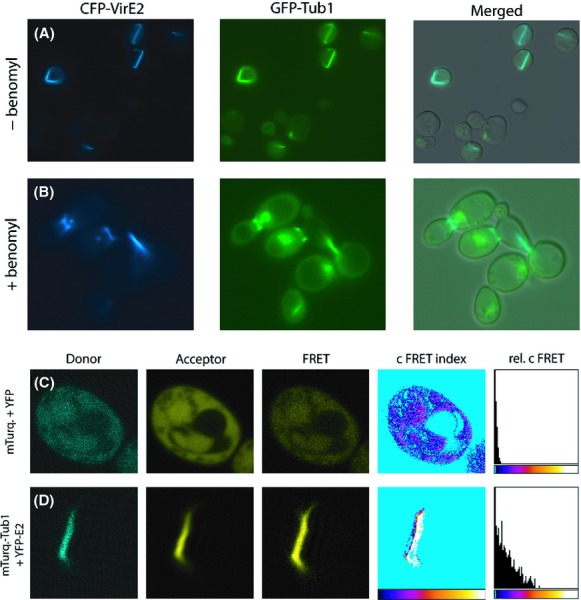
VirE2 colocalizes and physically interacts with Tub1p. (A) Confocal laser scanning microscopy of yeast strains MAS101-34CFP-VirE2 expressing CFP-VirE and GFP-Tub1. (B) Confocal laser scanning microscopy of yeast strains MAS101-34CFP-VirE2 treated for 45 min with 100 *μ*g/mL benomyl (C) FRET analysis on 426-34Turquoise-36YFP expressing free mTurquoise and free YFP. (D) FRET analysis on 426::Turquoise-TUB1[YFP-VirE2] expressing mTurquoise-Tub1p and YFP-VirE2. For FRET analysis the ImageJ plugin FRET and colocalization analyzer (Hachet-Haas et al. 2006) was used.

Prior to investigating VirE2 translocation from *Agrobacterium* to yeast we studied the localization of VirE2 fusions in yeast cells as has been done before in plant cells. To this end, we ectopically expressed YFP-VirE2, CFP-VirE2 and VirE2-GFP fusion proteins in the *Saccharomyces cerevisiae* strain CEN.pk113-3B under control of the *MET25* promoter and *CYC1* terminator. Microscopic studies showed that these VirE2 fusion proteins are stably expressed and present in thread-like structures within the yeast cells. Figure 2A and B show the subcellular localization of CFP-VirE2 and VirE2-GFP, respectively. This thread-like fluorescent signal was observed in more than 80% of more than 2000 fluorescent yeast cells studied, while in the remaining cells dot-like fluorescent structures were observed. The thread-like subcellular localization of VirE2 is remarkably similar to that of microtubules. In dividing yeast cells, microtubules are typically located between the two spindle poles (Moens and Rapport 1971). Expression of CFP-VirE2 in strain SHM284-1 (Pereira et al. 2001) expressing the spindle pole protein Spc42 C-terminally fused to Red Fluorescent Protein (RFP) showed that CFP-VirE2 localizes in the same way as microtubules between the two spindle poles of the mother and daughter cell during cell division (Fig. 2C). Expression of CFP-VirE2 in strain MAS101 which coexpresses a genomically integrated GFP-*TUB1* gene showed that CFP-VirE2 indeed colocalizes with GFP-Tub1p (Fig. 3A). In all cases where we observed both thread-like fluorescence of CFP-VirE2 and GFP-Tub1p, the VirE2-derived fluorescence colocalized with fluorescence from GFP-Tub1p. To investigate whether disruption of microtubules influences the localization of CFP-VirE2, we exposed yeast cells expressing GFP-Tub1p and CFP-VirE2 to a relatively high dose of the microtubule-destabilizing drug benomyl (Schatz et al. 1988) In this way at least 100 yeast cells were studied. Disruption of microtubule structures was observed ˜45 min after the addition of benomyl (Fig. 3B). At this time point both GFP-Tub1p and CFP-VirE2 filaments were broken. CFP-VirE2 still colocalizes with the remains of the GFP-Tub1p filaments (Fig. 3B).

**Figure 4:**
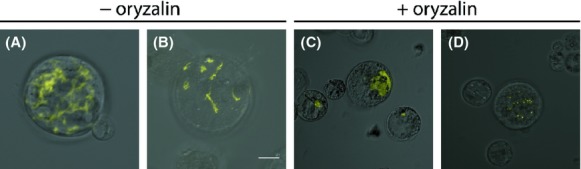
Confocal LSM microscopy of *Arabidopsis* Col-0 protoplasts expressing YFP-VirE2 and the effect of oryzalin treatment. YFP-VirE2 was expressed from a pART7 based vector under control of the 35S promoter and OCS terminator. (A and B), YFP-VirE2 localization in protoplasts. (C and D), YFP-VirE2 localization in protoplasts after 60 min of oryzalin treatment at 50 *μ*mol/L final concentrations. Scale bar, 12 *μ*m.

We found that a similar location is also possible in plant cells. When we transiently expressed YFP-VirE2 in *Arabidopsis* protoplasts thread-like structures of YFP-VirE2 were observed in the transformed protoplasts, comparable to those observed in yeast (Fig. 4A and B). To visualize the effect of microtubule disruption on the VirE2 localization, protoplasts were treated with oryzalin, a herbicide that destabilizes microtubular structures by strongly binding to tubulin monomers (Baskin et al. 1994). Approximately 1 h after the start of oryzalin treatment we saw a significant change in VirE2 localization, in line with our benomyl experiments in yeast. Thread-like YFP-VirE2 structures were either completely abolished (Fig. 4C) or severely shortened (Fig. 4D). The images shown in Figure 4 are representative of the extremes in phenotypes observed without or with oryzalin treatment, respectively.

Salman et al. (2005) have shown that VirE2 binds to microtubules in vitro. To obtain evidence for an interaction of VirE2 with tubulin in vivo, we analyzed the close association between mTurquoise-Tub1p and YFP-VirE2 by determining the energy transfer between the two fluorophores (FRET). Upon excitation of mTurquoise, due to FRET colocalization of both fluorescent proteins was indeed detected as fluorescence in the YFP emission channel (Fig. 3D, FRET). In contrast, only background fluorescence was measured in cells expressing free mTurquoise and free YFP (Fig. 3C). The results shown in these figures are representative for three independent FRET studies done on yeast cells expressing either mTurquoise and YFP (as shown in Fig. 3C) or mTurquoise-Tub1p and YFP-VirE2 (as shown in Fig. 3D). After correction for donor and acceptor BT a FRET index can be calculated. The FRET index was corrected for the level of the donor fluorophore resulting in a relative FRET index, shown as histograms in Figure 3C and D. This relative FRET index for mTurquoise-Tub1p–YFP-VirE2 pair was higher than that of the unbound mTurquise–YFP pair. The relative FRET index for the mTurquoise-VirE2–YFP-VirE2 interaction is 11.9 ± 9.0 (*n* = 384) (mean ± SD, for 384 positive pixels) compared to 1.8 ± 1.1 (*n* = 5801) (mean ± SD, for 5801 positive pixels) for the mTurquoise–YFP interaction. Using the Student's *t*-test the former index is significantly (*P* < 0.0001) higher than the latter one, signifying that VirE2 and Tub1 are in close proximity to each other and indicative of a physical interaction.

### Use of BiFC to visualize VirE2 delivery into yeast during AMT

**Figure 5:**
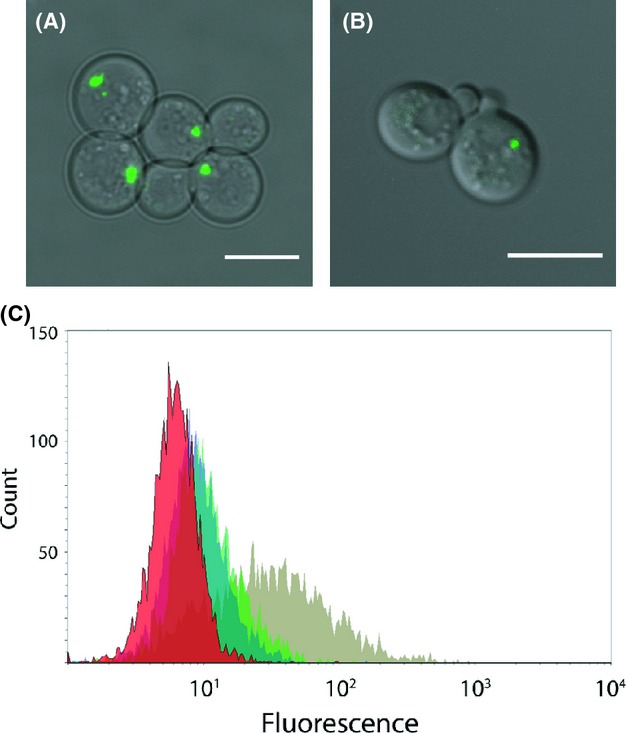
Visualization by BiFC of interactions between VirE2 proteins expressed in yeast. Confocal laser scanning microscopy micrographs of (A) 428-34VC[VirE2]/35VN[VirE2] cells expressing VC-VirE2 and VirE2-VN and (B) 428-34VN[VirE2]/36VC[VirE2] cells expressing VN-VirE2 and VC-VirE2. Scale bars: 7 *μ*m. (C) Flow cytometry histogram plot of CEN.PK113-3B (red), 428-34VC/35VN[VirE2] expressing VC and VirE2-VN (blue), 428-34VC[VirE2]/35VN expressing VC-VirE2 and VN (green) and 428-34VC[VirE2]/35VN[VirE2] expressing VC-VirE2 and VirE2-VN (olive).

Previous in vitro studies have shown that VirE2 can bind to other VirE2 molecules forming multi-protein structures (Frenkiel-Krispin et al. 2007). In order to investigate whether the interaction between VirE2 proteins can be used for visualization of VirE2 translocation, we first needed to ascertain that this interaction can be visualized in yeast by the BiFC approach (Nagai et al. 2002; Sung and Huh 2007). In cells expressing VirE2-VN (containing the N-terminal part of Venus, VN) and VC-VirE2 (containing the C-terminal part of Venus, VC) a clear fluorescent signal was seen using confocal microscopy, confirming the self-association between VirE2 proteins (Fig. 5A). In control cells with VirE2-VN and free VC or with free VN and VC-VirE2 hardly any detectable fluorescence was visible. Similarly, control cells with VN-VirE2 and free VC or with VirE2-VC and free VN showed next to no fluorescent signal (data not shown). Flow cytometry was used to confirm these results (Fig. 5C). We also tested the combination VN-VirE2 with VC-VirE2 (Fig. 5B) and VN-VirE2 with VirE2-VC (not shown). Although these interactions gave a detectable signal, the best signal was seen using cells expressing VC-VirE2 and VirE2-VN (Fig. 5A).

**Figure 6:**
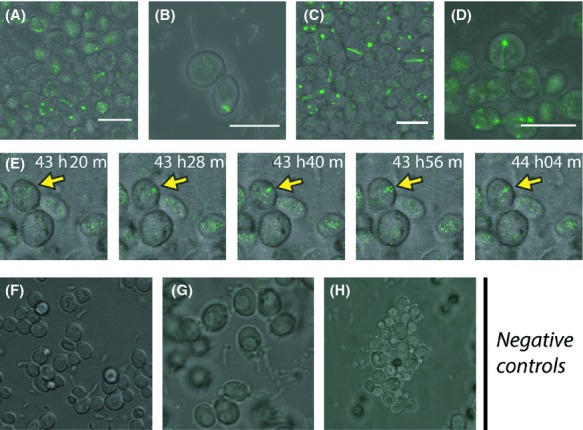
Visualization of VirE2 delivery into recipient yeast cells during cocultivation with *Agrobacterium tumefaciens* using BiFC. (A and B) Cocultivation of *A. tumefaciens* strain LBA2572(3163VC-VirE2) expressing VC-VirE2 and yeast strain 428-35VN[VirE2] expressing VirE2-VN; (C and D) Cocultivation of the T-DNA deficient *A. tumefaciens* strain LBA2573(3163VC-VirE2), expressing VC-VirE2 and yeast strain 428-35VN[VirE2], expressing VirE2-VN. (E) Time-lapse experiment of a cocultivation of LBA2572(3163VC-VirE2) and 428-35VN[VirE2] visualizing the delivery of VirE2 in host cells in vivo and in real time. The time-lapse reveals elongation of a thread-like structure over time in a recipient yeast cell (yellow arrow). (F) Negative control cocultivation of *A. tumefaciens* strain LBA1010 expressing untagged VirE2 and yeast strain 428-35VN[VirE2] expressing VirE2-VN; (G) Negative control cocultivation of the T-DNA deficient *A. tumefaciens* strain LBA1100 expressing untagged VirE2 and yeast strain 428-35VN[VirE2] expressing VirE2-VN; (H) Negative control cocultivation of the *virD4* mutant *A. tumefaciens* strain LBA2587(3163VC-VirE2) expressing VC-VirE2 and yeast strain 428-35VN[VirE2] expressing VirE2-VN. Scale bars: 7 *μ*m. YFP fluorescence resulting from BiFC is displayed green. The visible and YFP images were superimposed.

Vergunst et al. (2003) have shown that the C-terminal 50 amino acids of VirE2 are needed for translocation. To visualize delivery of VirE2 from *A. tumefaciens* into yeast during cocultivation, we therefore made use of *A. tumefaciens* strains expressing VirE2 N-terminally tagged with a part of Venus (VC-VirE2) and yeast expressing VirE2 tagged with the complementary part of Venus (VirE2-VN). We used *A. tumefaciens* donor strains lacking the native *virE*2 gene. All visualization studies described below were independently performed at least five times. As shown in Figure 6A and B, after 30 h of cocultivation clear fluorescent filamentous structures were visible in the recipient yeast cells, similar to those observed after expression of both VC-VirE2 and VirE2-VN in yeast (Fig. 5). Cocultivation mixtures of *A. tumefaciens* strains expressing VC-VirE2 and yeast strains expressing VirE2-VN were analyzed by microscopy every day for 6 days after the start of cocultivation and during the entire period filamentous structures were visible. The first fluorescent signal was visible 1 day after initiation of the cocultivation (data not shown). Comparable structures were found after cocultivation with donor strains containing or lacking T-DNA (Fig. 6C and D). In experiments using *A. tumefaciens* strains expressing VN-VirE2 and yeast strains expressing VirE2-VC, similar filamentous structures were visible, although with a somewhat lower intensity (data not shown). Negative control experiments were performed by cocultivating *A. tumefaciens* donors LBA1010 or LBA1100 that only express wild-type VirE2 with yeast expressing VirE2-VN and also by cocultivations of *Agrobacterium* expressing VC-VirE2 with yeast expressing only the complementary BiFC fragment VN not fused to VirE2. Also we verified that the signal seen was due to translocation by the T4SS by using a *virD4* mutant expressing VC-VirE2 as a donor with yeast expressing VirE2-VN. Control experiments did not yield any substantial fluorescent signals.

To visualize VirE2 delivery into yeast cells in real time, cells were taken from a cocultivation mixture of *A. tumefaciens* expressing VC-VirE2 and yeast expressing VirE2-VN 24–48 h after initiation of the cocultivation and observed by microscopy during an additional 10 h. Figure 6E shows images of a typical transformed yeast cell. Fluorescence intensity increased over a period of ˜20 min and the dot-like structure was elongated into a more filamentous structure in the next ˜25 min (Fig. 6E, yellow arrow).

### Use of the split GFP system to visualize VirE2 delivery by the T4SS

**Figure 7:**
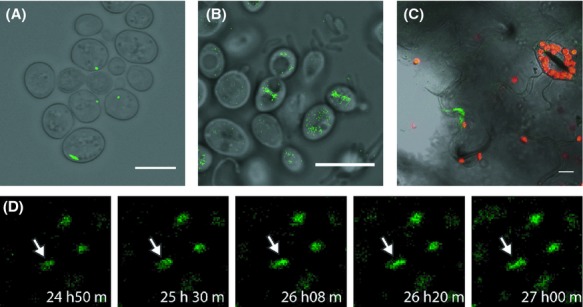
Visualization of VirE2 translocation from *Agrobacterium* to yeast and tobacco cells using the split GFP system. (A) Confocal LSM image of 426::GFP_1-10_-34GFP_11_[VirE2] cells expressing GFP 1-10 and GFP_11_-VirE2. Scale bar: 7 *μ*m. (B) Image of yeast strain 426::GFP_1-10_ after cocultivation with *Agrobacterium tumefaciens* strain LBA2573(3163GFP_11_-VirE2) for 42 h. Scale bar: 7 *μ*m. (C) Image of *Nicotiana tabacum* SR1 line expressing GFP 1-10 24 h after agroinfiltration with LBA2573(3163GFP 11-E2). Red, chlorophyll fluorescence. Scale bar, 12 *μ*m. (D) Time-lapse microscopy of a cocultivation of LBA2573(3163GFP_11_-E2) and yeast strain 426::GFP_1-10_. Co-cultivation times are indicated; h: hours and m: minutes.

Van Engelenburg and Palmer (2010) developed an elegant split GFP system by which they could visualize effector protein translocation from *Salmonella* into Hela cells by the T3SS. An advantage of this system is that also the delivery of effector proteins with no known interaction partners can be followed as the two GFP parts of this system spontaneously assemble. The major part embracing the first 10 strands of GFP (GFP 1-10) is expressed all over the cell so that the protein of interest fused with the small 13 amino acid 11th strand of the GFP *ß*-barrel solely contributes to the location of a reconstituted fluorescent signal. We first analyzed whether VirE2 can be visualized in yeast by the split GFP system. To this end, VirE2 was N-terminally tagged with GFP 11 and expressed together with GFP 1-10 in yeast. A reconstituted GFP signal was indeed detected. In corroboration with the results described above long and short VirE2 filaments were seen in the yeast cells (Fig. 7A).

To visualize VirE2 protein delivery from *A. tumefaciens* into yeast, cocultivations were performed with an *A. tumefaciens* strain expressing VirE2 N-terminally tagged with GFP 11 and the yeast recipient strain 426::GFP_1-10_ expressing GFP 1-10. After 42 h of cocultivation confocal microscopy showed a reconstituted GFP signal (Fig. 7B), indicating translocation of VirE2 from *A. tumefaciens* to yeast. Translocation was observed in roughly 1 per 1000 yeast cells, similar as observed using the BiFC method. Figure 7B shows that translocated VirE2 proteins are present in the recipient yeast cells in filaments or dots, similar as was observed after expression of GFP 11-VirE2 in yeast (Fig. 7A). This observation is in accordance with the observations made using BiFC (Fig. 6).

To visualize virulence protein delivery into host yeast cells in real time, cells were taken from a cocultivation mixture of *A. tumefaciens* expressing GFP 11-tagged virulence proteins and yeast expressing GFP 1-10 20 h after initiation of the cocultivation and observed by CLSM during an additional 16 h. Reconstituted GFP signal, resulting from VirE2 entry from *A. tumefaciens* into yeast cells, was first observed after 20–25 h of cocultivation in each of five independent experiments. Figure 7D (arrows) shows a fluorescent signal appearing which intensifies and is elongating into a filament during the next 90 min. This observation is comparable to that obtained using the BiFC approach to detect entry of VirE2 into yeast cells (Fig. 6E).

We also adopted the split GFP system to visualize virulence protein delivery into plant (tobacco) cells. To this end, we first constructed *N. tabacum* SR1 plants stably expressing GFP 1-10 from the 35S promoter and terminator. To visualize virulence protein translocation 4–5-week old SR1 plants expressing GFP 1-10 were infiltrated with *A. tumefaciens* strains expressing GFP 11-tagged VirE2. After 17–24 h the infiltrated leaves were analyzed for GFP fluorescence by confocal microscopy. Due to fluorescence originating from chlorophyll, even in untransformed plants fluorescence is detected in the GFP channel, making the detection of reconstituted GFP in plants more difficult than in yeast. After infiltration of the transgenic tobacco leaves with an *A. tumefaciens* strain expressing GFP 11-VirE2 fluorescent signals could be detected in leaf epidermal cells (Fig. 7D).

## Discussion

In this article, we describe new methodology for the direct visualization of protein translocation from pathogenic microbes into host cells by the T4SS in real time. To this end, we adopted split fluorophore tagging systems (Fig. 1) based on BiFC (Nagai et al. 2002; Sung and Huh 2007) and on the split GFP system (Ciruela 2008), respectively. An advantage of using a split fluorophore tagging system to visualize translocation of effector proteins is that neither the donor cells nor the recipient cells are fluorescent and, therefore fluorescence will only be detected after translocation of the effector proteins and reconstitution of the complete fluorophore in the host cells. The BiFC method has been used previously to visualize *Agrobacterium* effector protein–host protein interactions after coexpression in the plant host cells (Bhattacharjee et al. 2008). The split GFP system has not been used in T4SS so far, but was used earlier to visualize effector protein translocation into human cells by the T3SS of *Salmonella* (Van Engelenburg and Palmer 2010). In this article, we show that this system can equally well be employed to visualize translocation by the T4SS and in combination with both yeast and plant cells as recipient. The split GFP method is more versatile than BiFC for the visualization of effector protein translocation as it does not rely on prior knowledge of the interactions of the effector with other proteins in the host. We could use BiFC in the case of VirE2 as this protein self-associates in the absence of its chaperone VirE1 (Frenkiel-Krispin et al. 2007). We have also tested BiFC of VirE2 with VirE1, but this only led to a faint signal spread all over the cell and was therefore not used in cocultivation experiments. The self-association of VirE2 makes the fluorescent signal localized and strong. However, BiFC has also been used by us in the meantime to visualize translocation of other virulence proteins including VirE3 by using recipient cells with a tagged interaction partner, the pBrp protein in the case of VirE3 (P. A. Sakalis, G. P. H. van Heusden, and P. J. J. Hooykaas, unpubl. results).

Using both BiFC and the split GFP approach we were able to visualize translocation of VirE2 in vivo and in real time (Figs. 6E, 7D, respectively). Cocultivations with *A. tumefaciens* strains deleted for either *virB4* or *virD4* and expressing tagged VirE2 proteins did not result in any detectable fluorescent signal in recipient cells. Both *virB4* and *virD4* are essential for protein translocation by the T4SS confirming that translocation is mediated by the T4SS. The CRAfT assay has been used before to detect effector protein translocation by the *Agrobacterium* T4SS into both plant cells and yeast cells (Schrammeijer et al. 2003). The requirements for translocation were similar for both of these species. For visualization studies yeast has several advantages above plants cells. Yeast cells are transparent and do not contain large amounts of endogenous fluorescent compounds, similar to chlorophyll. Moreover, yeast strains stably expressing different combinations of tagged proteins can be constructed in much less time and effort than similar transgenic plant lines. Also, compared to yeast cells it was technically (more) difficult to perform time-lapse experiments with leaf segments of infiltrated tobacco plants to visualize protein translocation in real time. The plant epidermal cell is significantly larger than a yeast cell which makes it impossible to study many cells at the same time. Moreover, cells from a cut leaf segment might dehydrate or die during a time-lapse experiment, giving rise to autofluorescence which makes microscopic interpretation more difficult. Therefore, this work employed yeast to set up the system, but results were later confirmed in plants.

The split fluorophore tagging systems allows to follow the timing of translocation directly and also to determine the destination of the translocated proteins. We did not observe translocation of the VirE2 protein from *A. tumefaciens* into yeast and plant cells in less than ˜20 h of cocultivation. We have made similar observations when studying the translocation of other effector proteins including VirE3, VirF and VirD5, which have another subcellular localization, than VirE2 that is not linked to the microtubules (P. A. Sakalis, G. P. H. van Heusden, and P. J. J. Hooykaas, unpubl. results). In comparison, the observed period of time needed for the translocation of the SipA effector protein from *Salmonella* through a T3SS into human cells was considerably shorter. Using induced bacteria Schlumberger et al. (2005) observed that delivery began 10–90 sec after docking and proceeded for 100–600 sec until the bacterial SipA pool in the bacterium was depleted. Thus, time frames for delivery of proteins from prokaryotes into eukaryotic cells may greatly differ, depending on the host pathogen pair. The delay is not due to the time needed for reconstitution of the fluorophore as the work with *Salmonella* has shown that a fluorescent signal becomes visible within minutes after docking. As we used preinduced bacteria the delay in delivery must be due to later steps such as pilus formation, maturation or docking. T-DNA delivery to plant cells has been followed by the study of the kinetics of transcription of one of the genes on the T-DNA. By RT-PCR transcripts were detected only as early as 18–24 h after infection (Narasimhulu et al. 1996).

To study the subcellular localization of effector proteins in vivo, proteins are usually tagged with a fluorescent protein. However, it has been shown that localization based on ectopic expression of such fusion proteins may not reflect the natural situation. A number of studies have been published to reveal the localization of VirE2 tagged with a fluorophore inside the host cell. As reviewed by Gelvin (2010) the localization of VirE2 in the host cell is not unambiguous. Some studies report a cytoplasmic localization in plant cells (Bhattacharjee et al. 2008), while other studies show nuclear import of VirE2 (Citovsky et al. 1994). Bhattacharjee et al.(2008) reasoned that the different localizations reported could be due to different protein tags used in these studies which may alter the properties of the VirE2 protein. Nuclear localization of tagged VirE2 has been seen in tobacco, but has not been reported in *Arabidopsis* cells expressing this virulence protein. In our opinion it is therefore also possible that nuclear uptake of VirE2 is more efficient in tobacco than in *Arabidopsis* cells. This may depend on the abundance and status of the VIP1 protein, which has been shown to mediate VirE2 nuclear transport after activation by phosphorylation (Tzfira et al. 2001; Djamei et al. 2007). In human cells, which lack the VIP1 protein, VirE2 was found to be cytoplasmic and also for yeast a cytoplasmic localization was inferred from a genetic approach (Tzfira and Citovsky 2001).

In this study, we find localization of VirE2 at the cytoplasmic microtubules in both yeast and plant cells. This colocalization was confirmed by FRET analysis. These filaments were disrupted by compounds that disrupt microtubule, by benomyl in yeast and oryzalin in plant cells. Fluorescent filamentous structures became visible in the recipient yeast cells also after cocultivation of *A. tumefaciens* expressing VC-VirE2 with yeast expressing VirE2-VN (Fig. 3A) or after cocultivation of *A. tumefaciens* expressing GFP 11-VirE2 and yeast cells expressing GFP 1-10 (Fig. 7B). The fluorescent filamentous structures resembled those observed upon ectopic expression of both VC-VirE2 and VirE2-VN in yeast or after expression of CFP-or GFP-tagged VirE2 in yeast. Similar structures were also detected after cocultivation with a T-DNA deficient *A. tumefaciens* strain, indicating that this localization of VirE2 in the host cell does not depend on the presence of a transferred T-strand. Binding of VirE2 to the microtubules might point to a role of microtubule movement in the transfer of the T-strand toward the nucleus. There is some precedent for this as Salman et al. (2005) have shown that “animalized VirE2” is able to move along microtubules in vitro experiments with *Xenopus* cells. Further experimentation is needed to find out whether VirE2 (together with VIP1) uses the microtubular transport system for nuclear delivery of the T-complex.

## Supporting Information

**Data S1.** Plasmid constructions. **Table S1.** Yeast strains used in this study. **Table S2.** *Agrobacterium* strains used in this study. **Table S3.** Plasmids used in this study. **Table S4.** Primers used in this study.
