# Supplementary material for: Visualization of VirE2 protein translocation by the Agrobacterium type IV secretion system into host cells
Source: Microbiologyopen. 2013 Dec 27;3(1):104–17. doi: 10.1002/mbo3.152 (PMC3937733; doi:10.1002/mbo3.152)
Supplement: Supplementary file 1 — Data S1. Plasmid constructions. Table S1. Yeast strains used in this study. Table S2. Agrobacterium strains used in this study. Table S3. Plasmids used in this study. Table S4. Primers used in this study. [file mbo30003-0104-sd1.docx]

**SUPPORTING INFORMATION**

**Plasmid constructions.** All plasmids used and constructed in this study are listed in Table S3. Cloning steps were performed in *E. coli* strain DH5α. PCR amplifications were done with Phusion™ High-Fidelity DNA Polymerase and Table S4 lists all primers used for PCR amplifications.

The coding DNA sequence of *A. tumefaciens virE2* was PCR amplified from the pSDM3659 plasmid. A PCR fragment *Spe*I-VirE2-*Xma*I, amplified with *Spe*I-VirE2-Fw and *Xma*I-VirE2-Rev, was cloned into pJET1.2 generating pJET1.2[VirE2]. Subsequently, a *Spe*I-*Xma*I fragment with VirE2 was cloned into pUG34CFP and pUG36YFP to construct pUG34CFP[VirE2] and pUG36YFP[VirE2], respectively. PCR fragment *Spe*I-VirE2ΔTGA-*Xma*I, amplified with *Spe*I-VirE2-Fw and *Xma*I-VirE2ΔTGA-Rev, was cloned into pJET1.2 generating pJET1.2[VirE2ΔTGA]. The SpeI-XmaI fragment with VirE2ΔTGA was then cloned into pUG35 to make pUG35[VirE2]. VirE2 is N-terminally fused to CFP in pUG34CFP[VirE2] and to YFP in pUG36YFP[VirE2]. pUG35[VirE2] has a C-terminal fusion of VirE2 to GFP. Transcription of all VirE2 fusion proteins in plasmids in pUG34CFP[VirE2], pUG36YFP[VirE2] and pUG35[VirE2] is regulated by the *MET25* promoter and *CYC1* terminator.

For construction of BiFC plasmids we used the yeast tagging vectors EF210802 (pFA6a-VN-HIS3MX6) and EF210803 (pFA6a-VC-HIS3MX6) (Sung and Huh, 2007) as templates to PCR amplify the N- (VN) and C-terminal (VC) part of the coding DNA sequence from Venus (Nagai *et al.*, 2002). BiFC plasmids pUG34VN, pUG34VC, pUG36VN and pUG36VC were constructed through replacement of a *Xba*I-*Spe*I fragment with CFP (in case of pUG34CFP) or YFP (in case of pUG36 YFP) by *Xba*I-*Spe*I fragments with VC or VN. These plasmids were used to generate N-terminal fusions with VN or VC under control of the *MET25* promoter and *CYC1* terminator. *Xba*I-VN-*Spe*I was obtained by PCR using *Xba*I-F2-Fw and *Spe*I-VN-Rev, subsequently cloned into pJET1.2 making pJET1.2[VNn], finally the *Xba*I-VN-*Spe*I fragment was ligated into *Xba*I and *Spe*I digested pUG34CFP and pUG36, generating pUG34VN and pUG36VN respectively. Similarly VC was amplified with *Xba*I-F2-Fw and *Spe*I-VC-Rev, cloned into pJET1.2 making pJET1.2[VCn] and the *Xba*I-VC-*Spe*I fragment was cloned into *Xba*I and *Spe*I digested pUG34 and pUG36, generating pUG34VC and pUG36VC respectively.

BiFC plasmids pUG35VN and pUG35VC were constructed to make C-terminal fusions with the BiFC parts VN and VC. Both VN and VC were amplified using *Eco*RI-F2-Fw and *Eag*I-T_ADH1_-Rev and cloned into pJET1.2 making pJET1.2[VNc] and pJET1.2[VCc], respectively. Subsequently the *Eco*RI-VN-T_ADH1_-*Eag*I fragments were cloned into *Eco*RI and *Eag*I digested pUG35, replacing GFP by VN or VC and generating pUG35VN and pUG35VC. In these plasmids the original *CYC1* terminator is replaced by the *ADH1* terminator.

pUG34VN[VirE2] and pUG36VN[VirE2] were constructed by cloning an *Xba*I-VN-*Spe*I fragment from pJET1.2[VNn] into *Xba*I and *Spe*I digested pUG34CFP[VirE2] and pUG36YFP[VirE2], respectively. pUG34VC[VirE2] and pUG36VC[VirE2] were constructed by cloning an *Xba*I-VC-*Spe*I fragment from pJET1.2[VCn] into *Xba*I and *Spe*I digested pUG34[VirE2] and pUG36[VirE2], respectively. pUG35VN[VirE2] and pUG35VC[VirE2] were made by ligation of a *Spe*I-VirE2-*Xma*I fragment from pJET1.2[VirE2] into *Spe*I and *Xma*I digested pUG35VN and pUG35VC, respectively.

For FRET analysis additional plasmids pRS306-Turquoise-TUB1, pUG34Turquoise and pUG34YFP[VirE2] were constructed. To make FRET plasmids containing the coding sequence of mTurquoise we used plasmid pmTurquoise-C1 (Goedhart *et al.*, 2010) as template for PCR amplifications. PCR fragment *Xho*I-Turquoise-*Bam*HI, amplified with *Xho*I-mTurquoise-Fw and *Bam*HI-*Spe*I-Turquoise-Rev, was cloned into pJET1.2 generating pJET1.2[mTurquoise]. Plasmid pRS306-Turquoise-TUB1 was constructed by replacing the CFP encoding *Xho*I-*Bam*HI fragment in pRS306-CFP-TUB1 (Jensen *et al.*, 2001) with a *Xho*I-*Bam*HI fragment containing mTurquoise from pJET1.2[mTurquoise]. Finally, plasmid pUG34YFP[VirE2] was constructed by replacing the CFP encoding *Xba*I-*Spe*I fragment in pUG34CFP with the YFP encoding *Xba*I-*Spe*I fragment from pUG36YFP.

To express *virE2* tagged N-terminally with VN or VC in *A. tumefaciens* under control of the *virE* promoter, the plasmids pSDM3163[VN-VirE2] and pSDM3163[VC-VirE2] were made. Using the primers *Nde*I-F2-Fw and *Xma*I-VirE2-Rev, *Nde*I-VN-VirE2-*Xma*I and *Nde*I-VC-VirE2-*Xma*I fragments were obtained by PCR, using pUG34VN[VirE2] and pUG34VC[VirE2] respectively, as templates. These fragments were then ligated into pJET1.2, generating pJET1.2[VN-VirE2] and pJET1.2[VC-VirE2]. *Nde*I-*Xma*I fragments containing the DNA coding sequence for the tagged VirE2 proteins VN-VirE2 and VC-VirE2 were cloned into *Nde*I and *Xma*I digested pSDM3163 to generate vectors pSDM3163[VN-VirE2] and pSDM3163[VC-VirE2], respectively.

The coding DNA sequence of superfolder GFP 1-10 (Cabantous *et al.*, 2005) was amplified from vector pCMV-mGFP 1-10 Hyg Amp which was purchased from Sandia Biotech, Inc. An *Xba*I-GFP 1-10-*Xho*I PCR fragment, obtained by using *Xba*I-GFP_1-10_-Fw and *Xho*I-GFP_1-10_-Rev, was cloned into pJET1.2 generating pJET[GFP_1-10_]. Subsequently, an *Xba*I-*Xho*I fragment with GFP 1-10 was cloned into vector pUG34CFP*,* digested with *XbaI* and *Xho*I, replacing the CFP coding sequence by GFP 1-10 to create pUG34GFP_1-10_. Yeast integrative plasmid pRS305-GFP_1-10_ was constructed by insertion of a *Bsp*EI-*Eag*I fragment from pUG34GFP_1-10_, containing the GFP 1-10 sequence flanked by the *MET25* promoter and *CYC1* terminator sequences, into *Xma*I and *Eag*I digested pRS305 (Sikorski and Hieter, 1989). In both pUG34GFP_1-10_ and pRS305-GFP_1-10_ transcription of GFP 1-10 is regulated by the *MET25* promoter and *CYC1* terminator. pUG34GFP_11_[VirE2] was constructed by ligation of a *Spe*I-*Eag*I fragment with VirE2 from pUG34CFP[VirE2] into *Spe*I and *Eag*I digested pUG34GFP_11_.

A DNA fragment with the 48 bp coding sequences of GFP11 linked to a 27 bp linker (Kaddoum *et al.*, 2010), was obtained by annealing phosphorylated oligonucleotides *Xba*I-*Nde*I-GFP_11_-Fw and *Spe*I-GFP_11_-Rev. To this end, the oligonucleotides were mixed, boiled for 5 min and incubated at 37°C for 3 hrs. This fragment, which is flanked by *Xba*I and *Spe*I compatible overhangs, was inserted into *Xba*I and *Spe*I digested pUG34CFP, replacing the CFP coding sequence by the GFP_11_ and linker sequence to construct pUG34GFP_11_. This plasmid is used to tag proteins at their N-terminus with GFP 11, transcriptionally regulated by the *MET25* promoter and *CYC1* terminator.

Plasmid pSDM3163[GFP_11_-VirE2] was obtained by cloning an *Nde*I-*Hin*dIII fragment from pUG34GFP_11_[VirE2] containing the coding sequence for GFP 11 and the N-terminal 1090 bases coding for VirE2 into *Nde*I and *Hin*dIII digested pSDM3163, thereby replacing *virE1* by GFP 11. Expression of GFP11-*virE2* is under control of the vir*E* promoter.

To make transgenic tobacco SR-1 lines expressing GFP 1-10, plasmid pCambia1302-_GFP1-10_ was constructed. PCR amplification with *Nco*I-GFP_1-10_-Fw and *Bst*EII-GFP_1-10_-Rev and template pUG34GFP_1-10_ was carried out to obtain an *Nco*I-GFP_1-10_-*Bst*EII fragment. This fragment was cloned into pJET1.2 to produce pJET1.2[GFP_1-10_]2. Subsequently an *Nco*I-*Bst*EII fragment from pJET1.2[GFP_1-10_]2 coding for GFP 1-10 was ligated into pCambia1302 (Cambia Australia^®^), digested with *Nco*I and *Bst*EII, replacing the mGFP coding sequence by GFP 1-10 to create pCambia1302-GFP_1-10_.

All PCR fragments were verified by sequencing before using them for plasmid constructions. Correct ligation was checked by restriction analysis and sequencing.

**TABLE S1: Yeast strains used in this study**

| **Yeast strain** | **Genotype** | **Source / reference** |
| --- | --- | --- |
| CEN.PK113-3B | *MATalfa ura3-52 his3-delta1* | P. Kötter, Göttingen, Germany. |
| CEN.PK2-1C | *MATa ura3-52 leu2-112 trp1-289 his3-delta1* | P. Kötter, Göttingen, Germany. |
| MAS101  (AFS403) | *S. cerevisiae* pRS306[P_HIS3_-GFP-TUB1] (*URA3*) | A. W. Murray, U.S. (Straight, 1997) |
| SHM284-1 | *MATa ura3-52 leu2delta1*::*pSM976 trp1delta63 his3delta200 SPC42-RFP-KanMX6* | E. Schiebel, Heidelberg, Germany (Pereira *et al.*, 2001) |
| 428-34CFP-VirE2  (GG3342) | CEN.PK113-3B  pUG34[P_MET25_-CFP-VirE2-T_CYC1_] (*HIS3*) | This study |
| 428-36YFP-VirE2  (GG3343) | CEN.PK113-3B  pUG36[P_MET25_-YFP-VirE2-T_CYC1_] (*URA3*) | This study |
| 428-35-VirE2-GFP  (GG3344) | CEN.PK113-3B  pUG35[P_MET25_- VirE2-GFP-T_CYC1_] (*URA3*) | This study |
| MAS101-34CFP-VirE2  (GG3347) | MAS101  pUG34[P_MET25_-CFP-VirE2-T_CYC1_] (*HIS3*) | This study |
| 284-34CFP-VirE2  (GG3348) | SHM284-1 (Spc42p-RFP)  pUG34[P_MET25_-CFP-VirE2-T_CYC1_] (*HIS3*) | This study |
| 428-34VN[VirE2]/35VC  (GG3349) | CEN.PK113-3B  pUG34[P_MET25_-VN-VirE2-T_CYC1_] (*HIS3*) pUG35[P_MET25_-VC-T_ADH1_] (*URA3*) | This study |
| 428-34VN[VirE2]/36VC  (GG3350) | CEN.PK113-3B  pUG34[P_MET25_-VN-VirE2-T_CYC1_] (*HIS3*) pUG36[P_MET25_-VC-T_CYC1_] (*URA3*) | This study |
| 428-34VC[VirE2]/35VN  (GG3351) | CEN.PK113-3B  pUG34[P_MET25_-VC-VirE2-T_CYC1_] (*HIS3*) pUG35[P_MET25_-VN-T_ADH1_] (*URA3*) | This study |
| 428-34VC[VirE2]/36VN  (GG3352) | CEN.PK113-3B  pUG34[P_MET25_-VC-VirE2-T_CYC1_] (*HIS3*) pUG36[P_MET25_-VN-T_CYC1_] (*URA3*) | This study |
| 428-34VN[VirE2]/35VC[VirE2]  (GG3353) | CEN.PK113-3B  pUG34[P_MET25_-VN-VirE2-T_CYC1_] (*HIS3*) pUG35[P_MET25_-VirE2-VC-T_ADH1_] (*URA3*) | This study |
| 428-34VN[VirE2]/36VC[VirE2]  (GG3354) | CEN.PK113-3B  pUG34[P_MET25_-VN-VirE2-T_CYC1_] (*HIS3*) pUG36[P_MET25_-VC-VirE2-T_ADH1_] (*URA3*) | This study |
| 428-34VC[VirE2]/35VN[VirE2]  (GG3355) | CEN.PK113-3B  pUG34[P_MET25_-VC-VirE2-T_CYC1_] (*HIS3*) pUG35[P_MET25_-VirE2-VN-T_ADH1_] (*URA3*) | This study |
| 428-34VC[VirE2]/36VN[VirE2]  (GG3356) | CEN.PK113-3B  pUG34[P_MET25_-VC-VirE2-T_CYC1_] (*HIS3*) pUG36[P_MET25_-VN-VirE2-T_ADH1_] (*URA3*) | This study |
| 426-34Turquoise/36YFP  (GG3371) | CEN.PK2-1C  pUG34[P_MET25_-Turquoise-T_CYC1_] (*HIS3*) pUG36[P_MET25_-YFP-T_CYC1_] (*URA3*) | This study |
| 426-34Turquoise-VirE2  (GG3372) | CEN.PK2-1C  pUG34[P_MET25_-Turquoise-VirE2-T_CYC1_] (*HIS3*) | This study |
| 426-36YFP-VirE2  (GG3373) | CEN.PK2-1C  pUG36[P_MET25_-YFP-VirE2-T_CYC1_] (*URA3*) | This study |
| 426::Turquoise-TUB1/34YFP-VirE2  (GG3374) | CEN.PK2-1C  *ura3*::pRS306[P_HIS3_-Turquoise-TUB1-T_HIS3_] (*URA3*)  pUG34YFP (*HIS3*) | This study |
| 426::GFP_1-10_  (GG3388) | CEN.PK2-1C  *leu2*::pRS306[P_MET25_-GFP_1-10_-T_CYC1_] (*LEU2*) | This study |

**TABLE S2: Agrobacterium strains used in this study**

| **Agrobacterium strain** | **Specifications^a^** | **Source / reference** |
| --- | --- | --- |
| LBA1010 | C58 containing pTiB6, Rif | (Koekman *et al.*, 1982) |
| LBA1100 | C58 containing pTiB6Δ (ΔT-DNA, Δocc, Δtra), Rif, Spc | Beijersbergen *et al.*, 1992 |
| LBA1143  (LBA1100ΔB4) | *virB4* deletion in LBA1100, Rif, Spc, T4SS deficient | Beijersbergen *et al.*, 1992 |
| LBA2572  (LBA1010ΔE2) | *virE2* deletion in LBA1010, Rif | den Dulk-Ras and Hooykaas, unpublished |
| LBA2573  (LBA1100ΔE2) | *virE2* deletion in LBA1100, Rif, Spc | Hodges et al., 2006 |
| LBA2587  (LBA1100ΔD4) | *virD4* deletion in LBA1100, Rif, Spc, T4SS deficient | Hubber et al. ,2004 |
| LBA2572(3163VC-E2) | LBA2572 with pSDM3163[VC-VirE2]. Expression of the VC-VirE2 fusion protein under control of the *virE* promoter, Rif, Gm | This study |
| LBA2573(3163VC-E2) | LBA2573 with pSDM3163[VC-VirE2]. Expression of the VC-VirE2 fusion protein under control of the *virE* promoter, Rif, Spc, Gm | This study |
| LBA1143(3163VC-E2) | LBA1143 with pSDM3163[VC-VirE2]. Expression of the VC-VirE2 fusion protein under control of the *virE* promoter, Rif, Gm | This study |
| LBA1143(3163VN-E2) | LBA1143 with pSDM3163[VN-VirE2]. Expression of the VN-VirE2 fusion protein under control of the *virE* promoter, Rif, Gm | This study |
| LBA2587(3163VC-E2) | LBA2587 with pSDM3163[VC-VirE2]. Expression of the VC-VirE2 fusion protein under control of the *virE* promoter, Rif, Gm | This study |
| LBA2587(3163VN-E2) | LBA2587 with pSDM3163[VN-VirE2]. Expression of the VN-VirE2 fusion protein under control of the *virE* promoter, Rif, Gm | This study |
| LBA2572(3163GFP_11_-E2) | LBA2572 with pSDM3163[GFP_11_-VirE2]. Expression of the GFP 11-VirE2 fusion protein under control of the *virE* promoter, Rif, Gm | This study |
| LBA2573(3163GFP_11_-E2) | LBA2573 with pSDM3163[GFP_11_-VirE2]. Expression of the GFP 11-VirE2 fusion protein under control of the *virE* promoter, Rif, Spc, Gm | This study |

^a^ tra: transfer region, occ: octopine catabolism, Rif: rifampicin, Spc: spectinomycin, Km: kanamycin, Gm: gentamicin, Δ: deletion

**TABLE S3: Plasmids used in this study**

| **Name** | **Properties** | **Source / reference** |
| --- | --- | --- |
| pSDM3659 | pBBR6 with 4.266 kb *virE* operon | den Dulk-Ras and Hooykaas, unpublished |
| pUG34CFP  (pRUL1001) | Centromeric plasmid to make N-terminal CFP fusions under control of the *MET25* promoter and *CYC1* terminator. *HIS3* marker. | Miedema and van Heusden, unpublished |
| pUG35 | Centromeric plasmid to make C-terminal GFP fusions under control of the *MET25* promoter and *CYC1* terminator. *URA3* marker. | Güldener and Hegemann, unpublished |
| pUG36YFP  (pRUL1004) | Centromeric plasmid to make N-terminal YFP fusions under control of the *MET25* promoter and *CYC1* terminator. *URA3* marker. | Miedema and van Heusden, unpublished |
| pJET1.2 | CloneJET™ PCR Cloning pUC19 based vector for blunt cloning | Fermentas UAB |
| pJET1.2[VirE2]  (pRUL1236) | pJET1.2 with *virE2* flanked by *Spe*I and *Xma*I restriction sites. | This study |
| pJET1.2[VirE2ΔTGA]  (pRUL1237) | pJET1.2 with *virE2* without stop codon flanked by *Spe*I and *Xma*I restriction sites. | This study |
| pUG34YFP[VirE2]  (pRUL1239) | Centromeric plasmid with YFP-VirE2 under control of *MET25* promoter and *CYC1* terminator. *HIS3* marker. | This study |
| pUG34CFP[VirE2]  (pRUL1242) | Centromeric plasmid with CFP-VirE2 under control of *MET25* promoter and *CYC1* terminator. *HIS3* marker. | This study |
| pUG35[VirE2]  (pRUL1243) | Centromeric plasmid with VirE2-GFP under control of *MET25* promoter and *CYC1* terminator. *URA3* marker. | This study |
| pUG36YFP[VirE2]  (pRUL1244) | Centromeric plasmid with YFP-VirE2 under control of *MET25* promoter and *CYC1* terminator. *URA3* marker. | This study |
| pFA6a-VN-His3MX6  (EF210802) | Yeast tagging pFA6a based vector containing 560 bp N-terminal part of Venus (VN). | Sung and Huh, 2007 |
| pFA6a-VC-His3MX6  (EF210803) | Yeast tagging pFA6a based vector containing 300 bp C-terminal part of Venus (VC). | Sung and Huh, 2007 |
| pJET1.2[VNn]  (pRUL1246) | pJET1.2 with the N-terminal Venus part (VN) flanked by *Xba*I and *Spe*I restriction sites. The VN part is designed for N-terminal fusions. | This study |
| pJET1.2[VCn]  (pRUL1247) | pJET1.2 with the C-terminal Venus part (VC) flanked by *Xba*I and *Spe*I restriction sites. The VC part is designed for N-terminal fusions. | This study |
| pJET1.2[VNc]  (pRUL1248) | pJET1.2 with VN-TADH1 flanked by *Eco*RI and *Eag*I restriction sites. The VN part is designed for C-terminal fusions. | This study |
| pJET1.2[VCc]  (pRUL1249) | pJET1.2 with VC-TADH1 flanked by *Eco*RI and *Eag*I restriction sites. The VC part is designed for C-terminal fusions. | This study |
| pUG34VN  (pRUL1177) | Centromeric plasmid to make N-terminal fusions with the N-terminal Venus part under control of the *MET25* promoter and *CYC1* terminator. *HIS3* marker. | This study |
| pUG34VC  (pRUL1176) | Centromeric plasmid to make N-terminal fusions with the C-terminal Venus part under control of the *MET25* promoter and *CYC1* terminator. *HIS3* marker. | This study |
| pUG35VN  (pRUL1181) | Centromeric plasmid to make C-terminal fusions with the N-terminal Venus part under control of the *MET25* promoter and *ADH1* terminator. *URA3* marker. | This study |
| pUG35VC  (pRUL1180) | Centromeric plasmid to make C-terminal fusions with the N-terminal Venus part under control of the *MET25* promoter and *ADH1* terminator. *URA3* marker. | This study |
| pUG36VN  (pRUL1250) | Centromeric plasmid to make N-terminal fusions with the N-terminal Venus part under control of the *MET25* promoter and *CYC1* terminator. *URA3* marker. | This study |
| pUG36VC  (pRUL1251) | Centromeric plasmid to make N-terminal fusions with the C-terminal Venus part under control of the *MET25* promoter and *CYC1* terminator. *URA3* marker. | This study |
| pUG34VN[VirE2]  (pRUL1256) | Centromeric plasmid with *VN-virE2* under control of the *MET25* promoter and *CYC1* terminator. *HIS3* marker. | This study |
| pUG34VC[VirE2]  (pRUL1257) | Centromeric plasmid with *VC-virE2* under control of the *MET25* promoter and *CYC1* terminator. *HIS3* marker. | This study |
| pUG35VN[VirE2]  (pRUL1258) | Centromeric plasmid with *VirE2-VN* under control of the *MET25* promoter and *ADH1* terminator. *URA3* marker. | This study |
| pUG35VC[VirE2]  (pRUL1259) | Centromeric plasmid with *VirE2-VC* under control of the *MET25* promoter and *ADH1* terminator. *URA3* marker | This study |
| pUG36VN[VirE2]  (pRUL1260) | Centromeric plasmid with *VN-virE2* under control of the *MET25* promoter and *CYC1* terminator. *URA3* marker. | This study |
| pUG36VC[VirE2]  (pRUL1261) | Centromeric plasmid with *VC-virE2* under control of the *MET25* promoter and *CYC1* terminator. *URA3* marker. | This study |
| pSDM3163GFP11[VirE2]  (pSDM3756) | pSDM3163 based vector with coding sequence of GFP11-VirE2 under control of VirE promoter and terminator. | This study |
| pmTurquoise-C1 | Mammalian expression vector with *mTurquoise* | Goedhart *et al*.,2010 |
| pRS306-CFP-TUB1 | Yeast integrative vector with *CFP-TUB1* under control of the *HIS* promoter and terminator. *URA3* marker. | Jensen *et al*., 2001 |
| pJET1.2[Turquoise]  (pRUL1271) | pJET1.2 with *mTurquoise* flanked by *Xho*I and *Bam*HI restriction sites. | This study |
| pRS306-Turquoise-TUB1  (pRUL1273) | Yeast integrative vector with *Turquoise-TUB1* under control of the *HIS* promoter and terminator. *URA3* marker. | This study |
| pUG34Turquoise  (pRUL1274) | Centromeric plasmid to make N-terminal Turquoise fusions under control of the *MET25* promoter and *CYC1* terminator. *HIS3* marker. | This study |
| pUG34Turquoise[VirE2]  (pRUL1275) | Centromeric plasmid with Turquoise-VirE2 under control of *MET25* promoter and *CYC1* terminator. *HIS3* marker. | This study |
| pSDM3163 | pBBR6 with coding sequence of VirE1 and VirE2 under control of the VirE promoter | Schneider, unpublished |
| pJET1.2[VN-VirE2]  (pRUL1283) | pJET1.2 with VN*-virE2* flanked by *Nde*I and *Xma*I restriction sites. | This study |
| pJET1.2[VC-VirE2]  (pRUL1284) | pJET1.2 with VC*-virE2* flanked by *Nde*I and *Xma*I restriction sites. | This study |
| pSDM3163[VN-VirE2]  (pSDM3757) | pSDM3163 backbone with the coding sequence of VN-VirE2 under control of the VirE promoter | This study |
| pSDM3163[VC-VirE2]  (pSDM3758) | pSDM3163 backbone with the coding sequence of VC-VirE2 under control of the *virE* promoter | This study |
| pCambia1302 | High copy vector with *mGFP* under control of the *35S* promoter and the *CaMV* terminator. (bacterial kanamycin resitance, plant hygromycin selection) | Cambia, Australia^®^ |
| pCambia1302-GFP_1-10_  (pSDM3764) | pCambia1302 with GFP 1-10 under control of the *35S* promoter and the *CaMV* terminator. | This study |
| pART7-YFP  (pRUL1269) | pART7 based vector with *YFP* under control of the *35S* promoter and the octopine synthase (*OCS*) terminator. | Galvan Ampudia and Offringa, unpublished |
| pCMV-GFP 1-10 Hyg Amp | Plasmid containing coding sequence of mammalian-codon optimized GFP 1-10 D7 | Sand Biotech, Inc. (USA) |
| pJET1.2[GFP_1-10_] (pRUL1276) | pJET1.2 with GFP 1-10 flanked by *Xba*I and *Xho*I restriction sites. | This study |
| pUG34GFP_1-10_ (pRUL1277) | Centromeric plasmid with GFP 1-10 under control of the *MET25* promoter and *CYC1* terminator. *HIS3* marker. | This study |
| pRS305 | Yeast integration plasmid. *LEU2* selection marker. | Sikorski and Hieter, 1989 |
| pRS305-GFP_1-10_ (pRUL1278) | pRS305 containing GFP 1-10 under control of *MET25* promoter and *CYC1* terminator. *LEU2* marker, integration plasmid. | This study |
| pUG34GFP_11_ (pRUL1279) | Centromeric plasmid to make N-terminal GFP 11 fusions under control of the *MET25* promoter and *CYC1* terminator. *HIS3* marker. | This study |
| pUG34GFP_11_[VirE2] (pRUL1282) | Centromeric plasmid with GFP 11-VirE2 under control of *MET25* promoter and *CYC1* terminator. *HIS3* marker. | This study |
| pSDM3163[GFP_11_-VirE2] (pSDM3756) | pSDM3163 backbone with the coding sequence of GFP 11-VirE2 under control of the *virE* promoter | This study |
| pJET1.2[GFP1-10]2  (pRUL1299) | pJET1.2 with GFP 1-10 flanked by *Nco*I and *Bst*EII restriction sites. | This study |

**TABLE S4: Primers used in this study.**

| **Primer name** | **Sequence (5’ → 3’)^a,b^** |
| --- | --- |
| *Spe*I-VirE2 Fw | GGACTAGTATGGATCTTTCTGGCAA |
| *Xma*I-VirE2 Rev | CCCCCGGGTCAAAAGCTGTTGACGC |
| *Xma*I-VirE2ΔTGA Rev | CCCCCGGGAAAGCTGTTGACGCTTT |
| *Xba*I-F2 Fw | GCTCTAGAGGTCGACGGATCCCCGGGTT |
| *Spe*I-VN Rev | GGACTAGTAGTACCACCAGAACCCTCGATGTTGTGGCGGATC |
| *Spe*I-VC Rev | GGACTAGTAGTACCACCAGAACCCTTGTACAGCTCGTCCATG |
| *Eco*RI-F2 Fw | CGGAATTCGGTCGACGGATCCCCGGGTT |
| *Eag*I-T_ADH1_ Rev | GGCGGCCGGGCAAGCTAAACAGATCTA |
| *Xho*I-Turquoise Fw | CCCTCGAGATGGTGAGCAAGGGCGAGGA |
| *Bam*HI-*Spe*I-Turquoise Rev | AAGGATCCACTAGTCTTGTACAGCTCGTCCATGCC |
| *XbaI*-Turquoise Fw | AATCTAGAATGGTGAGCAAGGGCGA |
| *Nde*I-F2-Fw | CCCATATGGGTCGACGGATCCCCGGGTT |
| *Xma*I-VirE2-Rev | CCCCCGGGTCAAAAGCTGTTGACGC |
| *Nco*I-GFP1-10-Fw | GCCCATGGTTTCGAAAGGCGAGGA |
| *Bst*EII-GFP1-10-Rev | GGGTCACCTTATTTCTCGTTTGGGTCTT |
| *XbaI*-GFP1-10-Fw | GCTCTAGAATGGTTTCGAAAGGCGA |
| *XhoI*-GFP1-10-Rev | CCCTCGAGTTATTTCTCGTTTGGGT |
| *Xba*I-*Nde*I-GFP_11_-Fw | CTAGA CATATG GGGACCACATGGTGCTGCACGAGTACGTGAA CGCCGCCGGCATCACA ggcgacggcggcagcggcggcggcagc A |
| *Spe*I-GFP_11_-Rev | CTAGT gctgccgccgccgctgccgccgtcgcc TGTGATGCCGGCGGCGTT CACGTACTCGTGCAGCACCATGTGGTCCCGCATATGT |
| Leu2 1A | CAAGGATCTTACCGCTGTTG |
| Leu2 1S | AGAGGTCGCCTGACGCATAT |
| Leu2 2A | ACAACGACCAAGCTCACATC |
| Leu2 2S | ACTGGAACAACACTCAACCCTA |

a, restriction sites are underlined.

b, linker sequences are annotated in lowercase.

**REFERENCES SUPPORTING INFORMATION**

Beijersbergen, a, Dulk-Ras, a D., Schilperoort, R. a, and Hooykaas, P.J. (1992) Conjugative transfer by the virulence system of *Agrobacterium tumefaciens*. *Science* **256**: 1324–7.

Cabantous, S., Terwilliger, T.C., and Waldo, G.S. (2005) Protein tagging and detection with engineered self-assembling fragments of green fluorescent protein. *Nat Biotechnol* **23**: 102–7.

Goedhart, J., Weeren, L. Van, Hink, M.A., Vischer, N.O.E., Jalink, K., and Gadella, T.W.J. (2010) Bright cyan fluorescent protein variants identified by fluorescence lifetime screening. *Nature Methods* **7**: 137–139.

Hodges, L.D., Vergunst, A.C., Neal-McKinney, J., Dulk-Ras, Amke den, Moyer, D.M., Hooykaas, P.J.J., and Ream, Walt (2006) *Agrobacterium rhizogenes* GALLS protein contains domains for ATP binding, nuclear localization, and type IV secretion. *J Bacteriol* **188**: 8222–30.

Hubber, A., Vergunst, A.C., Sullivan, J.T., Hooykaas, P.J.J., and Ronson, C.W. (2004) Symbiotic phenotypes and translocated effector proteins of the *Mesorhizobium loti* strain R7A VirB/D4 type IV secretion system. *Mol Microbiol* **54**: 561–74.

Jensen, S., Segal, M., Clarke, D.J., and Reed, S.I. (2001) A novel role of the budding yeast separin Esp1 in anaphase spindle elongation: evidence that proper spindle association of Esp1 is regulated by Pds1. *The Journal of Cell Biology* **152**: 27–40.

Kaddoum, L., Magdeleine, E., Waldo, G.S., Joly, E., and Cabantous, S. (2010) One-step split GFP staining for sensitive protein detection and localization in mammalian cells. *BioTechniques* **49**: 727–736.

Koekman, B.P., Hooykaas, P.J.J., and Schilperoort, R.A. (1982) A functional map of the replicator region of the octopine Ti plasmid. *Plasmid* **7**: 119–132.

Moens, P.B., and Rapport, E. (1971) Spindles, spindle plaques, and meiosis in the yeast *Saccharomyces cerevisiae* (Hansen). *The Journal of Cell Biology* **50**: 344–61.

Nagai, T., Ibata, K., Park, E.S., Kubota, M., Mikoshiba, K., and Miyawaki, A. (2002) A variant of yellow fluorescent protein with fast and efficient maturation for cell-biological applications. *Nature Biotechnology* **20**: 87–90.

Pereira, G., Tanaka, T.U., Nasmyth, K., and Schiebel, E. (2001) Modes of spindle pole body inheritance and segregation of the Bfa1p-Bub2p checkpoint protein complex. *EMBO J* **20**: 6359–70.

Sikorski, R.S., and Hieter, P. (1989) A system of shuttle vectors and yeast host strains designed for efficient manipulation of DNA in *Saccharomyces cerevisiae*. *Genetics* **122**: 19–27.

Straight, F. (1997) Mitosis in living budding yeast: anaphase A but no metaphase plate. *Science (80- )* **277**: 574–578.

Sung, M., and Huh, W. (2007) Bimolecular fluorescence complementation analysis system for in vivo detection of protein – protein interaction in *Saccharomyces cerevisiae*. *Yeast* **24**: 767–775.
